# Cardioprotective Effects of Melatonin in Reperfusion Injury

**DOI:** 10.5935/abc.20180011

**Published:** 2018-01

**Authors:** Francisco R. M. Laurindo

**Affiliations:** Laboratório de Biologia Vascular - Instituto do Coração (InCor) - Faculdade de Medicina da Universidade de São Paulo, São Paulo, SP - Brazil

**Keywords:** Melatonin / pharmacology, Myocardial Reperfusion / physiopathology, Myocardial Reperfusion / prevention & control, Antioxidants / pharmacology

The possibility of limiting ischemic myocardial injury has been a major focus of cardiovascular research over 4 decades and literally thousands of interventions have been tested in this direction.^1^ Unfortunately, this has been a long, unsolved saga, and, apart from reperfusion, most interventions have been largely unsuccessful at the clinical arena.^1^ With the advent of clinical forms of reperfusion, the goal of these studies has slightly changed to identify adjuvant therapies that protect the myocardium from reperfusion injury. However, essentially no interventions have yet come to a realistic clinical testing scenario, although cellular therapies and other emerging interventions such as mitochondrial transplantation have concrete possibilities to bring a novel perspective to cardioprotection. The overall worldwide investments in cardioprotection research by funding agencies alone can be estimated to be over US$ 1 billion so far.^1^ Thus, it is plausible to ask whether it is reasonable to keep pursuing this type of investigation.^1^ To this end, the National Institutes of Health in the USA has created a consortium to perform a rigorous preclinical assessment of cardioprotective therapies (CAESAR).^2^ At present, the answer to these questions in the ischemia/reperfusion setting has yet to wait results of ongoing studies.^1,3^ Meanwhile, it is likely that understanding the mechanisms whereby specific interventions afford cardioprotection in reperfusion injury can have additional mechanistic implications in other areas. For example, several abnormalities of calcium handling observed in ischemia/reperfusion can be relevant to understand the pathophysiology of heart failure,^4^ and signaling pathways associated with hypoxia responses can modulate several aspects of vascular response to injury.^5^ Therefore, the investigation of nontoxic affordable interventions that provide cardiomyocyte protection, and in particular the identification of underlying associated mechanisms, can be relevant in diverse aspects.

The article by Hu S et al.,^6^ in this issue, brings a contribution to this scenario. The authors show that pharmacological concentrations of the pineal hormone melatonin (N-acetyl-5-metoxytriptamine) support cardioprotection. They first used an *in vitro* cardiomyocyte culture model submitted to hypoxia/reperfusion and showed that melatonin pretreatment results in decreased cell death and improved organization of the actin cytoskeleton. The authors interrogated whether these protective mechanisms could be associated with improved calcium handling. Indeed, melatonin incubation promoted decrease in cellular calcium overload and prevented the hypoxia/reperfusion-associated increase in the expression of the inositol trisphosphate receptor, as well the associated decrease in the expression of SERCA (sarcoplasmic reticulum calcium ATPase). The latter two alterations were reproduced in a rat model of ischemia/ reperfusion. Together, they indicate that the handling of calcium by the sarcoplasmic reticulum was improved, allowing the inference of a possible mechanism of cardioprotection. Of additional interest, melatonin incubation increased the phosphorylation of ERK1 (i.e., activation of the extracellular signal-regulated kinase 1) and pharmacological inhibition of ERK1 with the compound PD98059 negated the protective effects of melatonin on cell survival, actin organization and calcium handling. Thus, preservation of ERK1 activation is a likely mechanism of the protective effects of melatonin.

This study adds to other reports indicating a cardioprotective effect of melatonin by an array of antioxidant mechanisms,^7^ which include the preservation of mitochondrial integrity.^8^ It is likely that such antioxidant effect may have contributed to the improved calcium handling. Altogether, these data suggest that the mechanisms associated with melatonin-dependent cardioprotection deserve further investigation that may lead to the development of affordable non-toxic interventions. These, in turn, might have multiple implications, including showing that cardioprotection is not yet dead.^1^
